# Ecosystem services from wildlife harvests

**DOI:** 10.1093/biosci/biae039

**Published:** 2024-05-22

**Authors:** Jerrold L Belant, Abigail Bennett, Kenneth F Kellner, Maria del Mar Mancha-Cisneros

**Affiliations:** Wild Foods Institute, Department of Fisheries and Wildlife, Michigan State University, East Lansing, Michigan, United States; Wild Foods Institute, Department of Fisheries and Wildlife, Michigan State University, East Lansing, Michigan, United States; Wild Foods Institute, Department of Fisheries and Wildlife, Michigan State University, East Lansing, Michigan, United States; Wild Foods Institute, Department of Fisheries and Wildlife, Michigan State University, East Lansing, Michigan, United States

Assessing ecosystem services can help guide decision-making and cost–benefit analyses of research, policy, and investments in environmental and sustainable development worldwide (Fisher et al. 2009). Wildlife harvests have historically been critical to human food security (Weber et al. 2021). Although the relative contributions of these harvests to overall food and nutrition security have declined as the use of domesticated livestock has increased, wildlife harvests remain important to the food security of specific groups and contribute substantial protein to broader populations (Golden et al. 2011). In addition, multiple benefits of wildlife harvests beyond food security exist but are infrequently considered. Ecosystem services of wildlife harvests include provisioning (revenue), regulating (human–wildlife conflicts, climate, conservation, disease), and cultural (spiritual and religious values, recreational opportunities) services. Considering wildlife harvests within an ecosystem services framework can help guide more holistic thinking regarding wildlife science and management.

Economic benefits related to wildlife harvests are substantial. In 2022, estimated expenditures from recreational hunting in the United States was $45.2 billion (USFWS 2023). In Spain, estimated hunting expenditures during 2016 was €5.47 million and directly or indirectly supported 186,759 full-time equivalent jobs (Sánchez-Garcia et al. 2021). Similar incomes were generated from communal conservancies through harvested wildlife and from photo tourism in Namibia, with additional nonfinancial benefits (e.g., meat) from conservancies allowing hunting (Naidoo et al. 2016).

Wildlife harvests can also be used to mitigate negative economic or environmental impacts of some populations. For example, changes in land use and wildlife management have resulted in some species considered overabundant (e.g., ungulates in Europe; Carpio et al. 2021). White-tailed deer (*Odocoileus virginianus*) overabundance in the United States results in billions of dollars in losses annually through vehicle collisions alone (McShea 2012) and native plant biodiversity loss through herbivory (Reed et al. 2022). Because of the diverse situations in which overabundance occurs, context-dependent adaptive management is required, which can include hunting (Apollonio et al. 2017). In response, numerous US local jurisdictions (e.g., towns and cities), have altered ordinances to allow recreational harvests or professional culling to reduce white-tailed deer abundance. These efforts not only can mitigate damage caused by white-tailed deer and other species but provide additional hunter opportunities of which meat from harvested animals often is provided to food donation programs (e.g., D'Angelo et al. 2012).

Wildlife harvests have been used to mitigate disease prevalence. Increased harvests of male mule deer (*Odocoileus hemionus*) corresponded with lower prevalence of chronic wasting disease the following year (Conner et al. 2021). Increased hunting opportunities for white-tailed deer harvests and other management actions were used to reduce bovine tuberculosis prevalence in infected deer to undetectable levels in 5 years (Carstensen and DonCarlos 2011). However, effective disease management employing wildlife harvesting often requires an integrated and adaptive management framework that considers disease and wildlife host attributes (e.g., Mysterud et al. 2021).

Wildlife harvests can help to reduce anthropogenic greenhouse gas emissions. Domestic livestock represented about 14.5% of total anthropogenic emissions (Gerber et al. 2013) and recent trends toward reducing emissions consider the role of locally sourced food that includes wildlife harvests (Valle Nunes et al. 2019). Throughout much of the world, wild harvests are locally sourced and reduce emissions through reduced transportation, processing, and packaging costs (e.g., Johnson et al. 2021). Greenhouse gas emissions from some harvested wildlife species (e.g., nonruminants) are less than that from more typical commercial ruminant livestock (Wilson and Edwards 2008).

Use of wild harvests for food consumption can reduce the rate of land conversion to agricultural production. In South Africa, total revenues for properties managed for wildlife (hunting and phototourism) exceeded those of properties that raised livestock only, with meat production from top wildlife properties similar to that of livestock farms (Taylor et al. 2020). Reducing land conversion to human-dominated landscapes can also contribute to the evolving concept of rewilding that increasingly recognizes people as part of landscapes or systems, including wildlife harvests as a component or supplemental management tool (e.g., Perino et al. 2019, Duckett et al. 2022).

Longstanding benefits of wildlife harvests include values afforded through culture and tradition. Wild harvests are a traditional means of companionship through shared harvesting, food preparation, and food sharing among members within or outside families (Kraft et al. 2021). Wildlife harvests also are strongly tied to spirituality (Fernández-Llamazarea and Virtanen 2020) across cultures throughout the world (Neumann et al. 2022). Furthermore, personal values including environmentalism and appreciation of nature are important to many individuals that hunt wildlife (e.g., Radder and Bech-Larsen 2008). Wildlife harvests also contribute to indigenous people's well-being by sustaining livelihood, culture, and identity (Bélisle et al. 2021). Wildlife harvests provide opportunities to experience the outdoors with corresponding social and psychological benefits (Gigliotti and Metcalf 2016). In the United States, during 2022, more than 14.4 million hunters spent an estimated 241 million days hunting (USFWS 2023), and more than 7 million people hunt in Europe (Benko et al. 2020).

## Limitations and caveats

Wildlife harvests have numerous benefits to the global human population, but improved understanding of whether benefits are mediated by corresponding adverse effects is required. For example, many hunters travel long distances within or among countries for hunting opportunities. These additional transport costs could reduce the net benefits provided by wildlife harvests (Johnson et al. 2021).

As with all animal food sources, there are risks associated with consuming wildlife. Frequent consumption of wildlife harvested using lead ammunition can result in elevated lead intake (Gerofke et al. 2018). Additional considerations include food safety such as exposure to foodborne pathogens (Gaulin et al. 2020), public acceptance of wild meat (Corradini et al. 2022), and comprehensive approaches to market development (Needham et al. 2023).

Finally, sustainability of wildlife harvests clearly is paramount to maintaining population and species viability, overall biodiversity conservation, and associated ecosystem processes. Wildlife harvests can provide sustainable food, although unsustainable harvests have contributed to global biodiversity loss. In North America, species population extirpations (e.g., American bison, *Bison bison*) and extinctions (e.g., passenger pigeon, *Ectopistes migratorius*) occurred because of indiscriminate harvests (Brown 2022). However, a shift in North American wildlife management because of overharvests, including implementation of numerous state and federal laws, resulted in the North American Model of Wildlife Conservation (Organ et al. 2012), in turn facilitating numerous species recoveries with sustainable wildlife harvests. National and international legal frameworks and policies are generally established to ensure sustainable wildlife harvests, but effectiveness can vary and unsustainable harvests, particularly illegal harvests of wildlife, appear common (Lee et al. 2020).

## Recommendations

Having identified numerous ecosystem services, we offer several recommendations to consider regarding continued human benefits from wild harvests. Most importantly, data on wildlife abundance is lacking for many harvested wildlife species worldwide (e.g., Global South; Golden et al. 2011) and is not standardized across species and geographies, undermining requisite knowledge to ensure sustainable yields and understanding of ecosystem services. Understanding distributions of wildlife harvest and supply chains to consumers can improve efficiency on the basis of needs or demands. Greater knowledge of the nexus among conservation, humans, and wildlife harvests is critical to achieve policy-related goals and objectives. Development of strategies to further public awareness of wildlife harvests and their role in economies, conservation, and food security can improve the integration of wildlife harvests in policy discourse.

## Author Biography

Jerrold L. Belant (jbelant@msu.edu), Abigail Bennett, Kenneth F. Kellner, and Maria del Mar Mancha-Cisneros are affiliated with the Wild Foods Institute, in the Department of Fisheries and Wildlife at Michigan State University, in East Lansing, Michigan, in the United States.
